# Extracellular vesicles are associated with the systemic inflammation of patients with seropositive rheumatoid arthritis

**DOI:** 10.1038/s41598-018-36335-x

**Published:** 2018-12-17

**Authors:** Catalina Burbano, Mauricio Rojas, Carlos Muñoz-Vahos, Adriana Vanegas-García, Luis A. Correa, Gloria Vásquez, Diana Castaño

**Affiliations:** 1 Grupo de Inmunología Celular e Inmunogenética, Instituto de Investigaciones Médicas, Facultad de Medicina, Universidad de Antioquia UdeA, Calle 70 No 52-21, Medellín, Colombia; 2 Unidad de Citometría de Flujo, Sede de Investigación Universitaria, Universidad de Antioquia UdeA, Calle 70 No 52-21, Medellín, Colombia; 3 Sección de Reumatología, Hospital Universitario de San Vicente Fundación, Medellín, Colombia; 4 Sección de Dermatología, Departamento de Medicina Interna, Facultad de Medicina, Universidad de Antioquia, Laboratorio Clínico VID, Obra de la Congregación Mariana, Medellín, Colombia

## Abstract

Patients with rheumatoid arthritis (RA) and autoantibodies, such as rheumatoid factor and those against cyclic citrullinated peptides, are designated as seropositive and have a more severe disease with worse prognosis than seronegative RA patients. Understanding the factors that participate in systemic inflammation, in addition to articular commitment, would allow better treatment approaches for prevention of RA comorbidities and disease reactivation. We evaluated whether monocyte subsets and extracellular vesicles (EVs) could contribute to this phenomenon. Seropositive patients had higher levels of proinflammatory cytokines than those of seronegative patients and healthy controls (HCs); however, this systemic inflammatory profile was unrelated to disease activity. High frequencies of circulating EVs positive for IgG, IgM, CD41a, and citrulline, together with altered counts and receptor expression of intermediate monocytes, were associated with systemic inflammation in seropositive patients; these alterations were not observed in seronegative patients, which seem to be more similar to HCs. Additionally, the EVs from seropositive patients were able to activate mononuclear phagocytes *in vitro*, and induced proinflammatory cytokines that were comparable to the inflammatory response observed at the systemic level in seropositive RA patients; therefore, all of these factors may contribute to the greater disease severity that has been described in these patients.

## Introduction

Rheumatoid arthritis (RA) is characterized by a systemic inflammatory state that mainly affects joints but also other organs, such as skin, eyes, lung, and the cardiovascular system^1^. Two groups of patients with RA are clearly differentiated: the seropositive and seronegative type. Rheumatoid factor (RF), and antibodies against cyclic citrullinated peptides (anti-CCP) are hallmarks of patients with seropositive RA^2^. The presence of anti-CCP in patients with seropositive RA has been associated with more persistent inflammation, greater erosive joint damage, more severe clinical manifestations and worse prognosis than those in patients with seronegative RA^3^. The participation of RF in the disease’s development is not clear yet; however, it is proposed that this autoantibody potentiates the pathological effects of anti-CCP^4^. After antigen recognition by these autoantibodies, the immune complexes (ICs) formed can activate mononuclear phagocytes in circulation and joints, promoting the secretion of inflammatory mediators, such as TNF-α, IL-6, and IL-1β^5^. These cytokines are biomarkers of systemic inflammation in RA and contribute to the immunopathology of this disease because their blocking is associated with clinical improvement and remission^6–8^.

The understanding of which circulating factors contribute to the systemic inflammation in RA is essential to find more promising alternatives for the intervention and treatment of these patients. A high number of extracellular vesicles (EVs) have been detected in circulation and synovial fluid of RA patients^9,10^. The role of EVs in the pathogenesis of RA has begun to be elucidated; for example, these vesicles express autoantigens, such as citrullinated peptides (CPs), form ICs, and participate in the delivery of miRNA, and fibroblast-like synoviocytes activation^11^. EVs contribute in cell-to-cell communication and have a broad spectrum of effects on immune responses, mainly on monocytes and macrophages, and induce TNF-α, IL-6, and IL-1β production^11,12^.

Different cell components are involved in the development of inflammation, including neutrophils, mastocytes, T and B lymphocytes, and monocytes/macrophages. Activation of these cells leads to the production of cytokines and mediators responsible for inflammation. More specifically, monocytes/macrophages have been found to be activated in RA^13–15^, to massively infiltrate synovial membranes in RA^16,17^, and to produce TNF-α^17^. It has been shown in RA patients that circulating EVs forming ICs (EV-ICs)^10,18^ induce leukotriene production by neutrophils *in vitro*, which indicates that these vesicles may contribute to the inflammatory process. We propose that EVs can activate monocytes in circulation and contribute to the systemic inflammatory process in RA, similar to that observed in other systemic autoimmune diseases^19^. However, the effect of EVs on monocytes has not been previously associated with RA. Therefore, we evaluated whether patients with seropositive RA had any particular profile of circulating EVs and monocytes that could be associated with their systemic inflammatory response and assessed the direct effect of EVs and EV-ICs in mononuclear phagocytes.

## Results

### Systemic inflammatory response in seropositive patients is characterized by high serum levels of TNF-α, IL-6, and IL-1β

**Figure 1:**
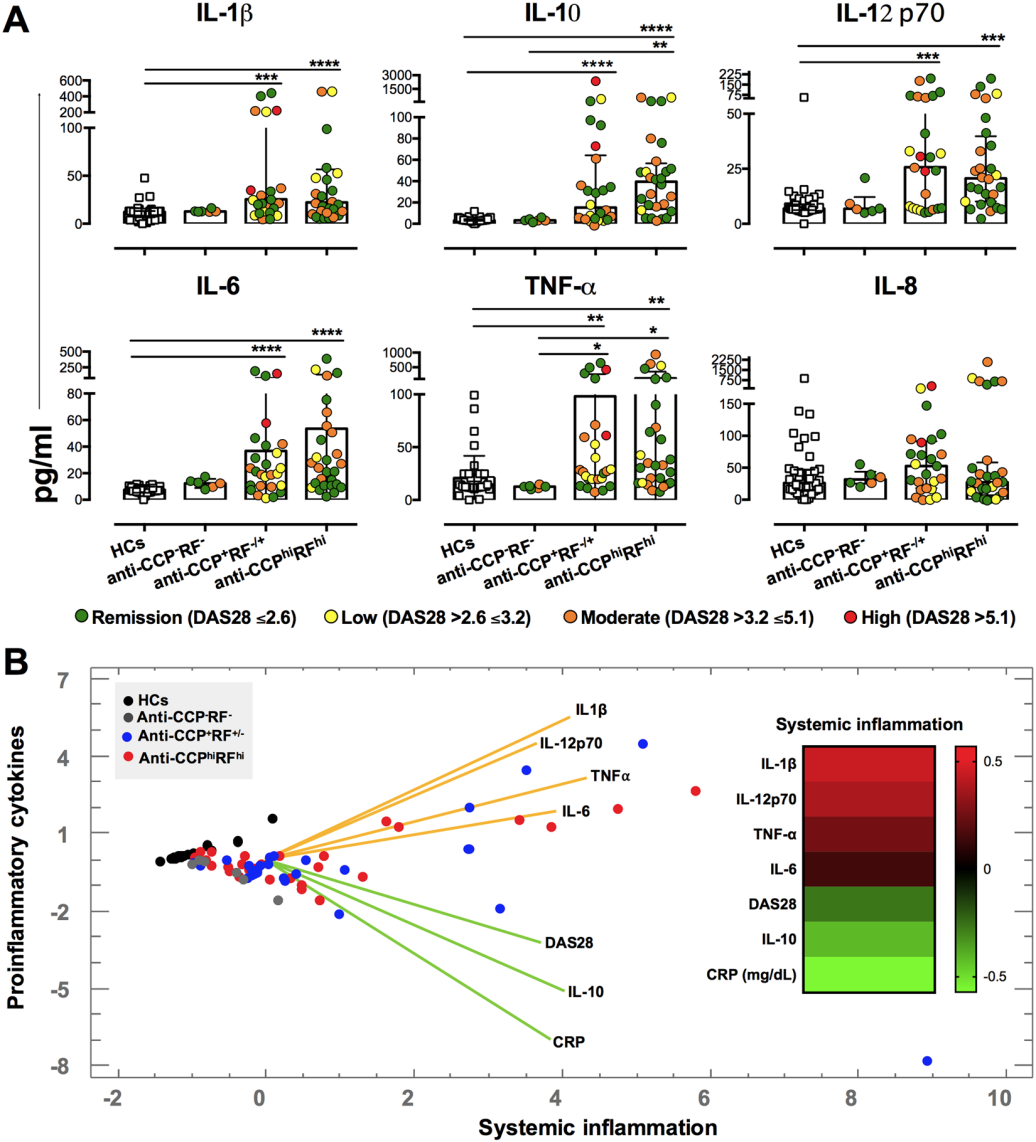
Seropositive RA patients (anti-CCP^+^RF^+/−^ and anti-CCP^hi^RF^hi^) have elevated levels of serum cytokines relative to those in seronegative patients and HCs. (**A**) Serum levels of IL-1β, IL-10, IL-12p70, IL-6, TNF-α, and IL-8 from RA patients (anti-CCP^−^RF^−^, anti-CCP^+^RF^+/−^, and anti-CCP^hi^RF^hi^) and HCs. Patients were classified according to DAS28 levels in Remission (DAS28 ≤ 2.6, green dots); Low activity (DAS28 > 2.6–≤ 3.2, yellow dots); Moderate activity (DAS28 > 3.2–≤ 5.1, orange dots); and High activity (DAS28 > 5.1, red dots). Comparisons among the groups were made by performing the Kruskal–Wallis test and Dunn’s *post-hoc* test. (**B**) PCA contrasting the proinflammatory cytokines (IL-1β, IL-12p70, IL-6, and TNF-α) and CRP, IL-10, and DAS28 variables in patients with seropositive and seronegative RA and HCs. The heat map inside PCA (right) shows the weights of each variable in component 2 (proinflammatory cytokines).

Alterations in serum cytokine levels have been described in different systemic inflammatory processes. Serum cytokines were measured as indicators of the systemic inflammatory state of RA patients. As shown in Fig. 1A, the classification of patients according to disease activity by disease activity score (DAS)−28 showed great variability in cytokine concentrations; patients classified under remission or low activity had either elevated or very low serum concentrations of cytokines. However, when patients were classified according to seropositivity, the groups of anti-CCP^+^RF^+/−^ and anti-CCP^hi^RF^hi^ (seropositive) patients had significantly elevated levels of TNF-α, IL-6, IL12p70, and IL-1β, but not IL-8, relative to those in healthy controls (HCs); anti-CCP^−^RF^−^ (seronegative) patients did not show such increases (Fig. 1A). Interestingly, when we evaluated the cytokines levels in patients with other systemic autoimmune disease (SADs), such as systemic lupus erythematosus (SLE), we did not find differences in proinflammatory cytokines serum concentrations compared with HCs (Data not shown). These findings indicate that serum cytokine concentrations are not increased in all SADs and may be characteristic of seropositive RA patients (anti-CCP^+^RF^+/−^ and anti-CCP^hi^RF^hi^).

A principal component analysis (PCA) was performed to define associations among serum cytokines (IL-1β, IL-12p70, IL-6, TNF-α, and IL-10), C-reactive protein (CRP), and DAS28 variables in patients with seropositive and seronegative RA and HCs (Fig. 1B). The PCA showed two components that explained the 65% of the variability and enabled separation of the variables between the two groups: a group associated with the proinflammatory cytokines that included most of the seropositive patients and a group associated with CRP, IL-10, and the DAS28 that was not clearly associated with seropositivity/seronegativity. HCs and anti-CCP^−^RF^−^ patients were not defined on the basis of these variables in either group and remained together. With the eigenvalues obtained from the PCA of each variable, a heat map was created. The heat map showed that the cytokines IL-1β, IL-12p70, TNF-α, and IL-6 defined seropositive patients better than did the DAS28 and CRP (Fig. 1B).

### High counts of intermediate monocytes in patients with seropositive RA

**Figure 2:**
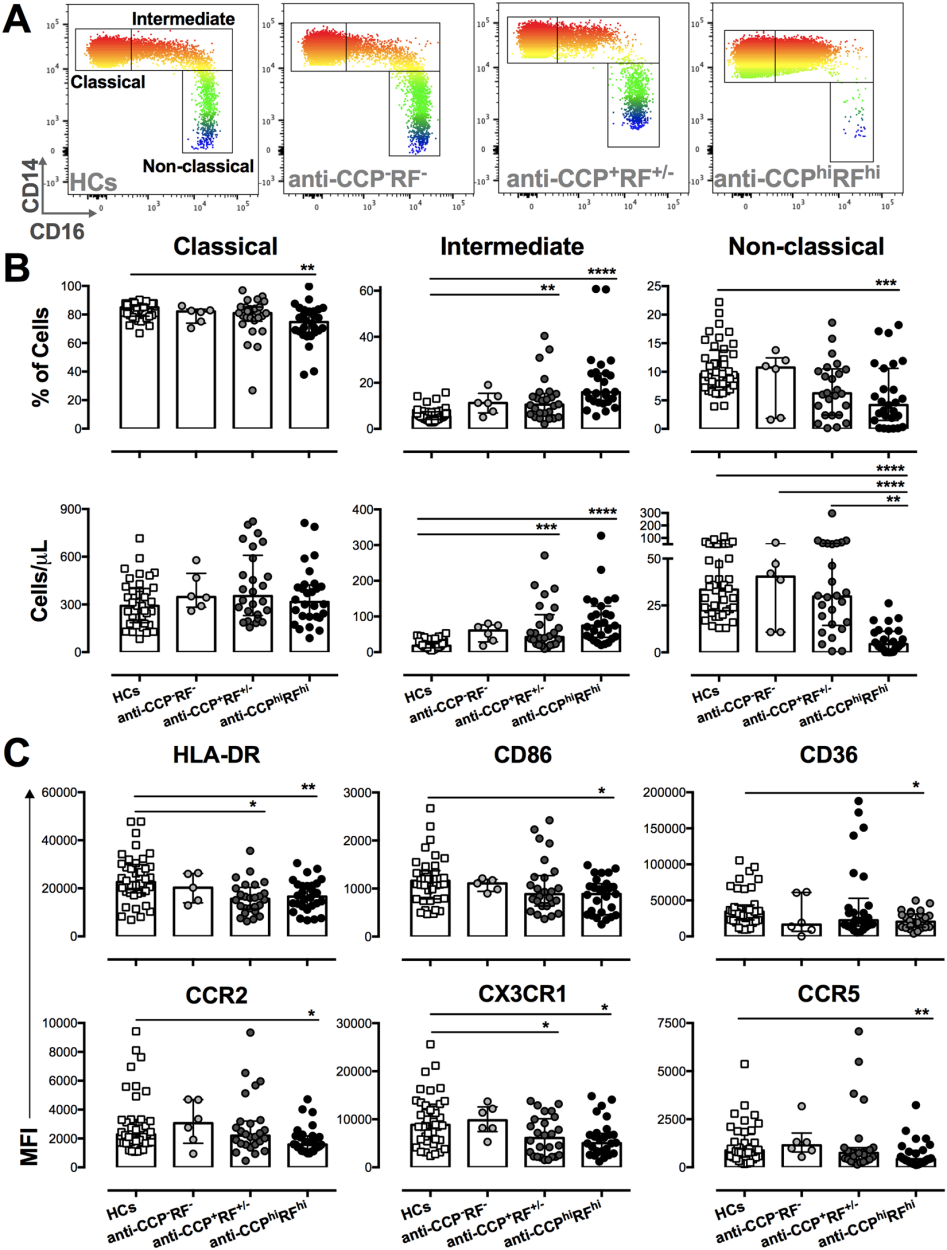
Seropositive patients had high counts of intermediate monocytes. (**A**) Representative CD14 and CD16 heat map plots of monocyte subsets (CD14++CD16− (classical), CD14++CD16+(intermediate), and CD14+CD16++ (non-classical) monocytes) gated on CD45+HLA-DR+ cells from the total blood of 1 individual of each study group. (**B**) Frequencies (upper panels) and absolute counts (lower panels) of classical, intermediate, and non-classical monocytes from anti-CCP^−^RF^−^, anti-CCP^+^RF^+/−^, and anti-CCP^hi^RF^hi^ RA patients, and HCs. (**C**) MFI of HLA-DR, CD86, CD36, CCR2, CX3CR1, and CCR5 on intermediate monocytes from anti-CCP^−^RF^−^, anti-CCP^+^RF^+/−^, anti-CCP^hi^RF^hi^ RA patients, and HCs. Comparisons among the groups were made by performing the Kruskal–Wallis test and Dunn’s *post-hoc* test.

Alterations in the frequency of circulating monocyte subsets^20^ and activation of total monocytes producing proinflammatory cytokines^13–15^ have been described in patients with RA. Therefore, we evaluated if the number, frequency, and phenotype of monocyte subsets were associated with seropositivity of patients with RA. A decrease in the proportion of classical monocytes was observed in anti-CCP^hi^RF^hi^ patients, which was not reflected in absolute counts (Fig. 2A,B). The intermediate monocytes were significantly elevated in both proportion and counts in the seropositive patients relative to HCs. Additionally, the non-classical monocytes were reduced in both the proportion and number in anti-CCP^hi^RF^hi^ patients (Fig. 2A,B). The expressions of receptors associated with the recognition of EVs and migration of monocyte subsets were evaluated in intermediate monocytes because these cells were the most affected in count and frequency in seropositive patients. Low expressions of HLA-DR and CX3CR1 in seropositive patients, and low expressions of CD86, CD36, CCR2, and CCR5 in anti-CCP^hi^RF^hi^ patients were observed compared with HCs (Fig. 2C). Low expression of CD18 was found in all monocyte subsets in seronegative patients relative to that in anti-CCP^hi^RF^hi^ patients and HCs (data not shown).

### EVs of seropositive patients are platelet-derived, CPs+, and form ICs

**Figure 3:**
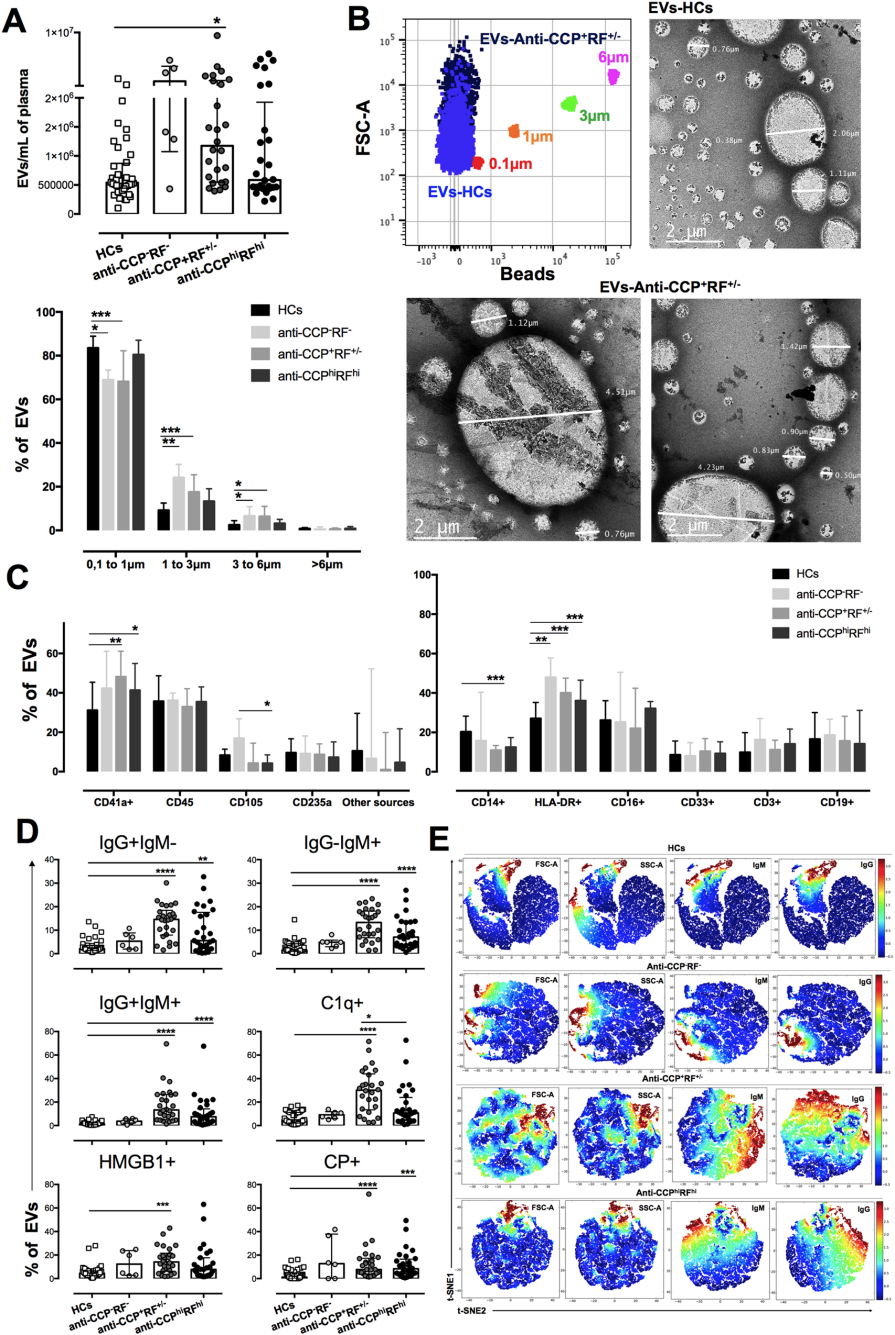
Circulating EVs of seropositive patients and HCs differ in number, cell sources, and phenotypes. Plasma EVs (**A**) count (above) and frequency of different sizes (below) in anti-CCP^−^RF^−^, anti-CCP^+^RF^+/−^, and anti-CCP^hi^RF^hi^ RA patients and HCs; (**B**) Left (above), representative dot plot of EVs in FSC-A of an anti-CCP^+^RF^+/−^ patient and a HC, with the intensity of Pacific blue beads of different sizes. Right (above), representative microphotographs of EVs from a HC and an anti-CCP^+^RF^+/−^ patient (below) using STEM. (**C**) Left: frequency of circulating EVs from different cellular sources: platelets (CD41a), leukocytes (CD45), endothelium (CD205), erythrocytes (CD235a), and other sources (EV negative for the evaluated molecules) from anti-CCP^−^RF^−^, anti-CCP^+^RF^+/−^, and anti-CCP^hi^RF^hi^ RA patients and HCs. Right: frequency of circulating EVs from different leukocyte sources in anti-CCP^−^RF^−^, anti-CCP^+^RF^+/−^, and anti-CCP^hi^RF^hi^ RA patients and HCs. (**D**) Frequency of circulating EVs positive to IgG + IgM-, IgG-IgM+, IgG+IgM+, C1q+, HMGB1+, and CP+ from anti-CCP^−^RF^−^, anti-CCP^+^RF^+/−^, and anti-CCP^hi^RF^hi^ RA patients and HCs. Comparisons among the study groups were made by performing the Kruskal–Wallis test and Dunn’s *post-hoc* test. (**E**) Collective t-SNE of EVs (1 × 10^5^) derived from five samples from each group analyzed and plotted (n = 20). Every dot represents a single EV, and the color indicates ArcSinh5-transformed expression values for each given marker analyzed and calculated over EVs from all samples varying from blue for lower expression to red for higher expression.

Recent reports indicate that EVs have a pivotal role in autoimmune diseases^21,22^ because of different pleiotropic effects on mononuclear phagocytes and other components of the immune system^21^. All patients with RA seem to display elevated EV counts; however, only anti-CCP^+^RF^+/−^ patients showed a significant difference compared with HCs (Fig. 3A). Regarding EV-size distribution, anti-CCP^−^RF^−^ and anti-CCP^+^RF^+/−^ patients had significantly decreased proportions of 0.1–1.0-µm EVs and elevated proportions of 1–3.0-µm and 3–6.0-µm EVs (Fig. 3A,B). The EV phenotype was also studied to identify the cellular source. The seropositive patients had elevated proportions of EV-CD41a+, and the anti-CCP^−^RF^−^ group had elevated EV-CD105+ relative to that of the anti-CCP^hi^RF^hi^ patients (Fig. 3C). Taking into account that approximately 50% of the EVs were derived from leukocytes (EV-CD45+), we evaluated different leukocyte sources. All groups of patients had elevated frequencies of EV-HLA-DR+, and the frequency of EV-CD14+ appeared to be decreased, but the difference was statistically significant only in the anti-CCP^+^RF^+/−^ group (Fig. 3C).

In addition to EV cell sources, components of EVs involved in the RA physiopathology were evaluated. The EV-ICs (IgG + IgM-, IgG-IgM+, and IgG + IgM+) and EV-CPs+ were significantly elevated in seropositive patients relative to those in HCs. The anti-CCP^+^RF^+/−^ patients had higher frequencies of EV-C1q+ and EV-High-mobility group box 1 (HMGB1)+ than those in the other groups; the EVs of seronegative patients were similar to those of HCs (Fig. 3D). The cell sources of EV-ICs, EV-CPs+, EV-C1q+, and EV-HMGB1+ were both leukocytes and platelets in total patients with RA; however, platelets were the main source in seropositive patients (data not shown). To illustrate the variations in ICs content of the EVs, 5 representative individuals of each group of patients with RA and HCs were randomly selected and a t-Distributed Stochastic Neighbor Embedding (t-SNE) analysis was performed. As expected, it was observed that vesicles from patients with seropositive RA had higher frequencies and a wider distribution of EV-IgM+ and EV-IgG+ than those of HCs and seronegative patients (Fig. 3E).

### EVs and monocyte subsets from synovial fluid and circulation share some characteristics

**Figure 4:**
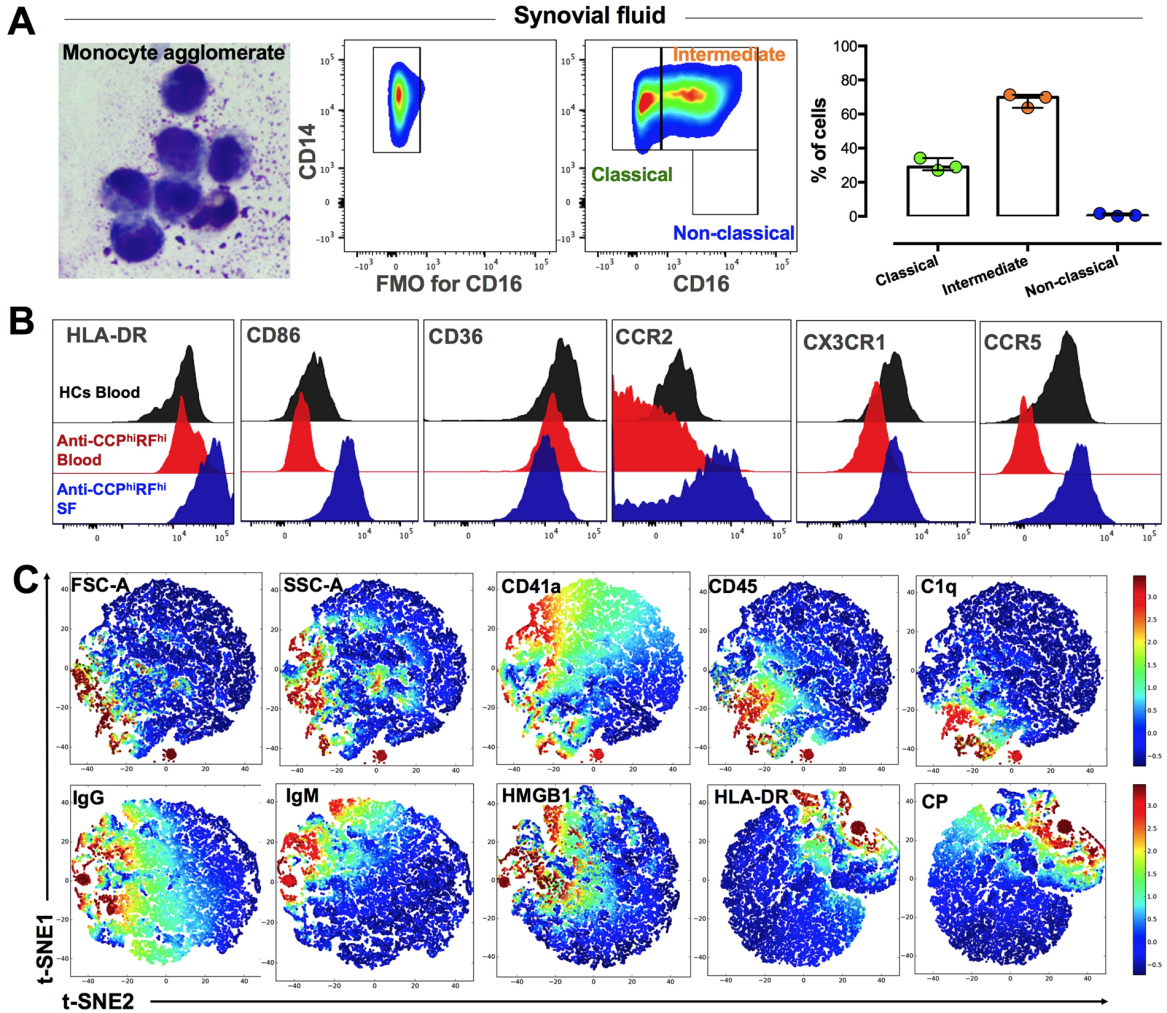
EVs and monocytes of seropositive patients exhibit some similar characteristics between SF and blood. (**A**) From left to right; a picture depicting a monocyte agglomerate in SF from an anti-CCP^hi^RF^hi^ patient. Representative CD14 and FMO for CD16 pseudo-color plots of monocyte subsets gated on CD45+HLA-DR+ cells from the SF of an anti-CCP^hi^RF^hi^ patient. Monocyte subset frequencies in the SF from three seropositive patients. (**B**) Representative histograms of HLA-DR, CD86, CD36, CCR2, CX3CR1, and CCR5 of intermediate monocytes from the blood of a HC (gray), an anti-CCP^hi^RF^hi^ patient (red), and the SF from the same patient (blue). (**C**) Collective t-SNE of EVs (1 × 10^5^) derived from three samples of SF from seropositive RA patients analyzed and plotted. Every dot represents a single EV and the color indicates ArcSinh5-transformed expression values for the given parameter analyzed and calculated over EVs from all samples varying from blue for lower expression to red for higher expression.

To evaluate whether the phenotype of EVs and monocyte subsets observed in circulation of seropositive patients also can be found in the synovial fluid (SF) of these patients, the SF of one anti-CCP^+^RF^+/−^ and two anti-CCP^hi^RF^hi^ patients were assessed. Approximately 20% of the SF infiltrating cells corresponded to monocytes, and strikingly, 70% of these cells corresponded to intermediate monocytes (Fig. 4A). However, there was a greater expression per cell of HLA-DR, CD86, CCR2, CX3CR1, CCR5, and CD32 but lower expression per cell of CD36, CD11b, and FcμR in intermediate monocytes of SF than those in circulating cells of the same patients (Fig. 4B and data not shown). Interestingly, mononuclear phagocytes from patients with seropositive RA of a 24 h culture showed higher expression of CD32 and lesser expressions of CD11b, CD36, and FcμR than those in HCs (Supplementary Figure 1–S1). Then, the phagocytes observed in the SF of patients could acquire this phenotype once they begin their process of tissue differentiation^23^.

Regarding EVs of SF and similar to circulating EVs, platelets and leukocytes were the main sources of these vesicles; also, the EVs of greater size (FSC-A) and complexity (SSC-A) were those that mainly form ICs in SF; these EVs also contain HMGB1, HLA-DR, and CP in different proportions (Fig. 4C).

### EVs, intermediate monocytes, and proinflammatory cytokines allow the discrimination of patients with RA according to seropositivity

**Figure 5:**
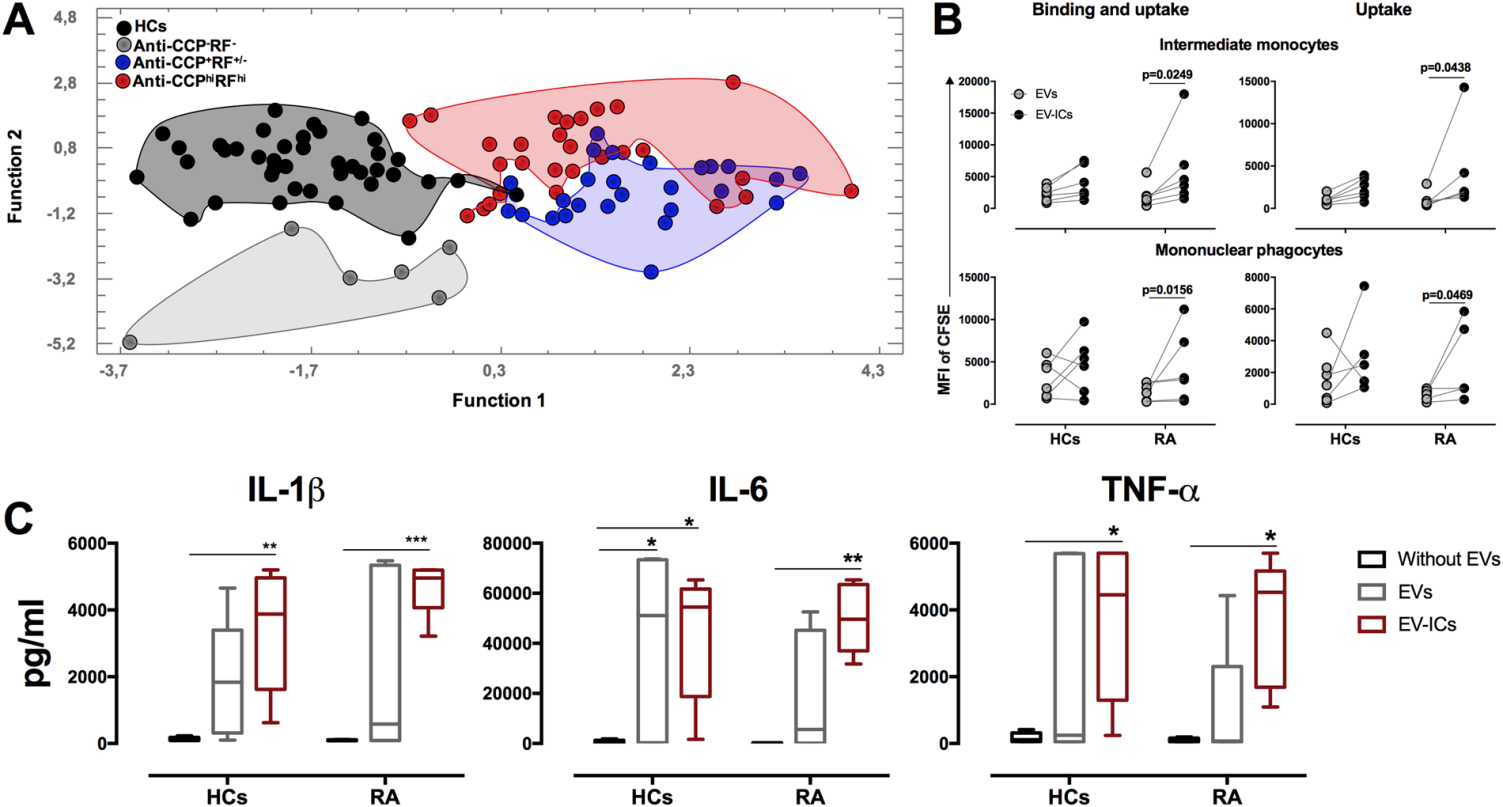
Variables of EVs, intermediate monocytes, and proinflammatory cytokines allow the discrimination of seropositive and seronegative patients with RA, whereas EVs induce phagocyte mononuclear activation. (**A**) Discriminant analysis was performed with 23 variables that included intermediate monocyte counts and receptors MFI (CD16, CD64, CD32, CD86, HLA-DR, CCR2, CCR5, CX3CR1), CD18 of three monocyte subsets, serum proinflammatory cytokines (IL-6, IL1β, and TNF-α) and frequencies of EVs positive to IgG, IgM, HMGB1, CP, CD41a, and HLA-DR from anti-CCP^−^RF^−^ (cloud of gray circles), anti-CCP^+^RF^+/−^ (cloud of blue circles), and anti-CCP^hi^RF^hi^ (cloud of red circles) RA patients and HCs (cloud of black circles). (**B**) Binding and uptake (left panels) and uptake (right panels) of EVs and EVs-ICs (3:1 vesicle: cell ratio) by intermediate monocytes (above) and mononuclear phagocytes cultured for 24 h from HCs and seropositive patients. Comparisons between the groups were performed by using the Wilcoxon signed-rank test (n = 7). (**C**) Levels of IL-1β, IL-6, and TNF-α in supernatants of mononuclear phagocytes from HCs and patients with RA cultured for 120 h in the presence or absence of EVs and EVs-ICs (3:1 vesicle: cell ratio). Comparisons between the groups were performed by using two-way ANOVA and Bonferroni’s *post-hoc* test (n = 7).

To establish which of the variables evaluated in monocyte subsets and EVs from blood are associated with autoantibody levels and systemic inflammation in RA patients, a discriminant analysis was performed with samples of patients and HCs. Twenty-three predictor variables that included intermediate monocyte counts and receptors (CD16, CD64, CD32, CD86, HLA-DR, CCR2, CCR5, CX3CR1), CD18 of three monocyte subsets, cytokines (IL-6, IL1β, and TNF-α), and frequencies of EVs positive for IgG, IgM, HMGB1, CPs, CD41a, and HLA-DR were included. The four study groups were significantly discriminated by these variables (p < 0.05), with a confidence level of 95%. However, anti-CCP^hi^RF^hi^ and anti-CCP^+^RF^+/−^ patients showed slight overlap. Interestingly, although HCs and anti-CCP^−^RF^−^ segregated to the same side, they were mutually excluded (Fig. 5A), which suggested that EVs and intermediate monocytes are closely associated with the systemic inflammation observed in seropositive patients.

### EVs from seropositive patients induce proinflammatory cytokine production by mononuclear phagocytes

To evaluate whether EVs from seropositive patients induce production of the cytokines related to the systemic inflammation detected in seropositive patients, we first evaluated the binding and internalization of these vesicles. Intermediate monocytes and mononuclear phagocytes of a 24 h culture from RA patients bound and internalized higher levels of EV-ICs than those of un-opsonized EVs (Fig. 5B). Finally, we found that in response to EVs, mainly EV-ICs, there were accumulations of IL-1β, IL-6, and TNF-α in the supernatants of mononuclear phagocytes of RA patients and HCs (Fig. 5C), which suggested that mononuclear phagocytes, after their EVs interaction, could contribute to the systemic inflammation observed in seropositive patients.

## Discussion

Multiple studies have identified several circulating cytokines and chemokines that reflect the systemic inflammation in RA^24,25^. However, little is known about the components responsible for this. We observed that higher levels of the serum cytokines IL-1β, IL-12p70, IL-6, and TNF-α were characteristic of patients with seropositive RA; in contrast, seronegative and SADs patients did not have elevated levels of these cytokines. The seropositive patients also exhibited high frequencies of circulating EVs positive for CD41a, IgG, IgM, and CPs, and altered counts, frequencies, and receptor expressions of monocyte subsets, mainly intermediate ones. These changes were not observed in seronegative patients, who more closely resembled HCs. Actually, EV-ICs from seropositive patients, more than EVs, induced the production of IL-1β, IL-6, and TNF-α by mononuclear phagocytes of RA patients and HCs *in vitro*. No one of the alterations observed in intermediate monocytes and EVs from RA patients were associated with disease activity evaluated by DAS28 (Figure S2 and data not shown). Therefore, these results together with the discriminant analysis suggest that in RA, the seropositive patients are those who mainly present systemic inflammation, which seems to be partially explained by the effect of EVs on mononuclear phagocytes. It is currently believed that seropositive and seronegative are two different RA entities, each with its own pathogenesis, disease course, and genes involved^26^. Our results agree with this hypothesis because seropositive patients, who have more severe disease and worse outcomes^27^, exhibited evident systemic inflammation.

Different circulating components, such as cytokines, EVs, and monocyte subsets, were identified according to the seropositivity status of RA patients. A few studies have analyzed IL-17, IL-15^28^, and IL-33^29^ cytokines according to anti-CCP positivity. Other studies have focused on evaluating cytokines in SF; Gómez-Puerta *et al*. in 2013^3^ found higher levels of cytokines IL-1β, IL-10, and IL-17 and the CCL20 chemokine in anti-CCP+ patients than those in seronegative patients. However, it was not clear if there was an association between the local and systemic inflammation process in those patients. Recent evidence has suggested the essential role of cytokines in anti-citrullinated protein antibody (ACPA)-positive patients with RA. It has been observed that the levels of IL-6, IL-17, and IL-8 were significantly elevated in SF from synovial ACPA-positive patients, and that the levels of IL-8 positively correlated with neutrophil counts and worse clinical manifestations in patients with RA^30^. Another study showed that exposure of osteoclast to ACPAs leads to a preferential release of IL-8, which mediates an autocrine activation of these cells^31^. Although we did not find significant differences in the serum levels of IL-8 among seropositive patients versus HCs and seronegative patients, it is important to note that we evaluated the serum and not SF concentrations of this cytokine. Therefore, it is possible that IL-8 participates in joint rather than in systemic inflammation. Additionally, it is noteworthy that IL-8 has been more often related to the initial hit of autoantibodies in RA, wherein this cytokine seems to be associated with the induction of initial bone resorption and with higher neuropathic pain than with pannus formation and inflammatory pain^32,33^.

Our results suggest that systemic inflammatory process does not always coincide with articular inflammation, as shown by the weak association between circulating cytokines and DAS28. This suggest that classifying the disease as active based only on the articular commitment of DAS28^2^ is insufficient to determine individuals with systemic and non-joint inflammation. In addition, we observed that not all patients with high DAS28 exhibited high levels of circulating inflammatory cytokines. This could be party explained by the generation of articular pain unrelated to a systemic inflammatory process because the joints’ sensory neurons can trigger pain in RA in the absence of local inflammation, tissue damage, or infection^34^. Additionally, irreversible destructive joint lesions often developed in the course of RA, remaining tender even after the inflammatory process subsides. Therefore, DAS28 is an objective score that may lead to an overestimation of disease activity because it could be significantly affected by the parameter of tender joint count. Clinically evaluating patients with RA based only in DAS28 may therefore lead to an inaccurate reflection of the total disease activity (articular and systemic).

Previously, it has been shown that *in vitro*, EV-ICs caused marked upregulation of leukotriene-B4 production by neutrophils^10^; however, several studies have shown that monocyte/macrophage are the major source of proinflammatory cytokines and chemokines in the inflamed RA joints, including TNF-α and IL-1β^35^. Here we observed that EVs, particularly EV-ICs from RA patients, induced IL-6, IL-1β, and TNF-α production by mononuclear phagocytes from HCs and RA patients. Although the inflammatory process in the joints could contribute to circulating cytokines^36^, our data suggest that other circulating sources of these mediators seem to exist in seropositive patients; particularly, our data support that EV-ICs and their interaction with monocytes could be contributing to this phenomenon. However, it is still matter of investigation whether or not monocytes are the main source of proinflammatory cytokines in seropositive patients at the circulation level.

Circulating cytokines and altered EVs are in a position to affect the function of distant tissues, including adipose, skeletal muscle, liver, and vascular endothelium^37,38^. It has been shown that chronic elevation in cytokine levels, irrespective of magnitude or cause, can generate a spectrum of proatherogenic changes that include insulin resistance, dyslipidemia, prothrombotic effects, pro-oxidative stress, and endothelial dysfunction in RA patients^25^. The ability to define the systemic inflammation in these patients could allow better establishment of the type and dose of the treatment to prevent comorbidities associated with seropositive RA and/or avoid disease reactivation. Therefore, the evaluation of serum cytokines, as well as some variables found in EVs and monocytes, could be a promising tool for follow-up of patients. Additionally, the identification of systemic inflammation in RA patients at the remission state defined by DAS28, could provide significant insights into the pathogenesis of RA and could be used to guide the initiation of disease modifying drugs in these patients.

In agreement with previous studies^9,10^, we found that platelets constitute the most frequent source of EVs in patients with RA; in addition, our study showed that this occurs mainly in seropositive patients. Furthermore, these patients had the highest frequencies of circulating EV-ICs+ and EV-CPs+, which indicates that these structures are an important source of ICs, autoantigens, and DAMPs in the circulation and SF of patients with RA, as previously proposed by others^10,18,21,22^. Depletion of platelets, in a deficient collagen receptor glycoprotein-(GP)VIa mouse model of RA, attenuated inflammatory arthritis, produced lower numbers of platelets-derived EVs *in vivo*, with minor ankle thickness and poor bone and cartilage erosion relative to those in control mice^9^, which suggested that these platelet-derived vesicles have an important role in the pathogenesis of arthritis.

In anti-CCP+RF+/− patients versus HCs a significant decrease in EV-CD14+ was observed. CD14 can be found in two forms; a form anchored to the membrane by a glycosylphosphatidylinositol tail (mCD14) and a soluble form (sCD14)^39^. It has been described that under inflammatory conditions, CD14 can be released from monocytes membrane into the culture milieu^40^. Therefore, the decrease frequency of this molecule observed in EVs from seropositive patients with RA could be based on the aforementioned phenomenon. In fact, an increase in the sCD14 blood level in patients with RA has been reported^41^. Moreover, an increase in the frequency of EV-HLA-DR+ was observed in all groups of patients with RA, together with high frequencies of platelet-derived EVs. Although the expression of HLA-DR in platelets or platelets-derived EVs has not been observed in healthy individuals, it has been described previously in platelets of patients with autoimmune diseases such as acute idiopathic thrombocytopenic purpura^42^. A study with similar patients showed that platelets-derived EVs also have HLA-DR expression^43^. Thus, the increase in EV-HLA-DR+ in patients with RA can either be related to an increased expression of HLA-DR+ in EV-CD41a+ or could correspond to a leukocyte source. Further experiments are required to solve these assumptions in patients with RA.

Similar to our results, Nathalie Cloutier *et al*.^10^ reported that circulating and SF EVs from patients with RA are highly heterogeneous in size and larger than those from HCs. However, according the t-SNE diagrams, no clear association was observed between the EV-size increase and the formation of ICs in seropositive patients with RA. In this respect, it is important to mention that we did not exclude EV aggregation, as previously reported^44^. Actually, it is striking that seropositive patients with RA have lower EVs count than seronegative ones. Although we do not have a clear explanation for this finding, we think it is possible that the formation of ICs by these vesicles can cause these structures to aggregate^44–46^; ICs activate and induce platelet aggregation^47^, and a considerable frequency of platelets-derived EVs in blood of patients with RA are CD62P+ and CD40L+ (platelet activation markers) (*Villar-Vesga J*, *et al*. *Submitted manuscript*). Therefore, whether ICs can promote aggregation of EVs in a manner similar to platelets remains unknown. Additional studies are required to corroborate this hypothesis. Previously, it was verified that EVs from the SF of RA patients form ICs through the interaction of anti-CCP with citrullinated autoantigens^10^. Although we did not explore this in detail here, our results agree with this because t-SNE analysis suggests that some EV-CPs+also were IgG+ and IgM+, mainly in seropositive patients.

In concordance with our study, other groups have demonstrated an increase in intermediate monocytes in the circulation of patients with RA^35^. Interestingly, we also found this increase was present mainly in the circulation and SF of seropositive patients and that EV-ICs were mostly captured by intermediate monocytes of RA patients compared with cells of HCs. Other monocyte subsets have been studied in seropositive patients with RA. In a recent study, two populations of circulating monocytes were evaluated^48^. M1 monocytes were defined as positive for CD14, CD68, and CCR2, whereas M2 monocytes were defined as positive for CD14, CX3CR1, and CD163. The authors demonstrated that patients with RA positive for ACPA had higher M1/M2 ratios. Moreover, a positive correlation was observed between the M1/M2 ratio and the number of differentiated osteoclast detected *in vitro* in samples of patients with RA. Furthermore, M1 monocytes produced higher concentrations of IL-6 upon stimulation with lipopolysaccharide than M2 monocytes. However, because the expression of CD16 was not studied in M1 and M2 monocytes, it is not possible at this point to compare these data with our results.

Previously, CD16+ monocytes found in the SF of RA patients have been associated with elevated levels of proinflammatory cytokines and joint destruction^17,49^, and RA patients have shown a negative correlation between intermediate monocyte frequency and disease activity scores; however, in our study we did not find this association (Figure S2 and data not shown), instead, we found that alterations in intermediate monocytes and EVs, along with the elevated serum concentrations of IL-6, TNF-α, and IL-1β, were closely related in seropositive patients. Actually, intermediate monocytes exhibited more remarkable phenotypic changes in the SF of seropositive patients with RA than those observed in the peripheral blood of the same patients. This suggests that intermediate monocytes in the joint have a more significant involvement than their blood counterparts, which can be related to proinflammatory activation or differentiation of these cells once they reach this location^50^. Additionally, EVs induced the secretion of IL1β, IL6, and TNF-α by mononuclear phagocytes. Previously, it has been proposed that the targeting of monocytes/macrophages should be a powerful way of inhibiting inflammation and bone erosion in RA^35^. Considering all of these aspects, it is tempting to speculate that the suppression of monocyte subsets, mainly intermediate ones (for example, by avoiding their interaction with EVs), may ameliorate the systemic inflammatory process observed in patients with seropositive RA. Actually, the anti-TNF-α (inflixamab, adalimumab, etanercept, among others) and anti-IL-6 (tocilizumab, sarilumab, sirukumab, among others) biological therapies, which are effective RA treatments^51,52^, may be partially useful by targeting monocyte proinflammatory responses to circulating EVs. Given that, in the present study, biological therapy was a variable of exclusion, further investigations are required to identify whether seropositive patients with RA under anti-TNF-α and anti-IL-6 treatments have fewer EVs and monocytes alterations, possibly related with less systemic inflammation. However, it is important to mention that the treatment received by the patients of this study was not related to the alterations observed in EVs and monocyte subsets (Data not shown).

Finally, our results in part explain the systemic inflammatory phenomenon that is occurring in patients with seropositive RA in which EVs and monocyte subsets appear to contribute. Seropositive patients are characterized by an increased number of intermediate monocytes and the presence of EV-ICs+ and EV-CPs+ together with the proinflammatory cytokines IL-1β, IL-6, and TNF-α. Therefore, the measurement of these circulating components could be a useful approach for establishing parameters of systemic inflammation in patients with RA that are not evaluated in any way by conventional tools, such as the DAS28.

## Methods

### Reagents, materials, and antibodies

RPMI-1640 medium supplemented with GlutaMAX, Dulbecco’s phosphate-buffered saline (DPBS) and fetal bovine serum (FBS) were purchased from Gibco-BRL (Grand Island, NY). Paraformaldehyde was acquired from Thermo Fisher Scientific, Inc. (Pittsburgh, PA). Histopaque®−1077, trypan blue, penicillin, and streptomycin were purchased from Cambrex-BioWhittaker (Walkersville, MD). Annexin-V, Annexin-V binding buffer, Lysing Solution, the BD^TM^ Human Inflammatory Cytometric Bead Array (CBA), and BD FACSFlow^TM^ were purchased from BD Pharmingen (San Diego, CA). Sterile polystyrene 12 × 75-mm tubes were acquired from BD Falcon (San Diego, CA). Absolute counting beads were obtained from Beckman Coulter (Hialeah, FL). A Fluoresbrite Calibration Grade Size Range Kit (YG calibration grade spheres with diameters of 0.5 μm, 1.0 μm, 2.0 μm, 3.0 μm, and 6.0 μm), Fluoresbrite YG Microspheres (0.1 μm), and Fluoresbrite YG Microspheres (0.2 μm) were acquired from Polysciences, Inc. (Warrington, PA). Rosette Sep Human Monocyte Enrichment Cocktail was obtained from STEMCELL Technologies (British Columbia, Vancouver, Canada).

Monoclonal anti-human MY4 (CD14)-FITC (Clone 322A-1) and MY4-RD1 (Clone 322A-1) were obtained from Beckman Coulter; the antibodies against human CD16-V450 (Clone 3G8), CD32-PE (Clone 3D3), CD36-APC (Clone CD38, also known as NL07), CD11b-PE (Clone D12), CD18-APC (Clone 6.7), FcμR-Alexa Fluor 647 (Clone HM14–1), CD41a-PE (Clone HIP8), CD3-PE (Clone 17A1), CD33-PB (Clone WM53), CD105-BV421 (Clone 266), CD19-V450 (Clone HIB19), CCR2-Alexa Fluor 647 (Clone 48607), and CD45-PE-Cy7 (Clone HI30) were acquired from BD Biosciences (San Diego, CA). Monoclonal anti-human CX3CR1-PE (Clone 2A9-1), CCR5-PE-Cy7 (Clone J418F1), HLA-DR-APC-Cy7 (Clone L243), were obtained from BioLegend (San Diego, CA). Monoclonal anti-human C1q-FITC (Clone ab4223), citrulline-primary antibody (Clone ab100932), and anti-rabbit IgG H&L-Alexa Fluor 647-secondary antibody (Clone ab150079) were obtained from Abcam (San Francisco, CA). The antibody against human HMGB1-APC (Clone 115603) from R&D Systems (Minneapolis, MN). The F(ab’)_2_ anti-IgG fragment conjugated with Alexa Fluor 488 and F(ab’)_2_ anti-IgM fragment conjugated with Alexa Fluor 647 were purchased from Jackson ImmunoResearch (Baltimore Pike, West Grove, PA). Anti-human CD235a-APC (Clone HIR2) was bought from eBioscience (San Diego, CA).

### Patients and controls

Sixty patients with RA, diagnosed according to the American College of Rheumatology criteria and European League Against Rheumatism criteria^2^, were recruited at the Rheumatology Service of the Hospital Universitario San Vicente Fundación (HUSVF, Medellin-Colombia); their median age was 55 years with a range of 30 to 77 years, and 84% were women. The patients were clustered in three groups: one seronegative [anti-CCP^−^RF^−^, (n = 6)] and two seropositive [anti-CCP^+^RF^+/−^ (n = 26) and anti-CCP^hi^RF^hi^ (n = 28)]; none of the patients received biological therapy. As controls, we included 40 HCs with a median age of 46 years with a range of 25 to 77 years, and 85% were women; and 60 SLE patients with a median age of 46 with a range of 25 to 77 years, and 85% were women, only for measurement of serum cytokines^19^. Our study was carried out in accordance with the Declaration of Helsinki. Research protocol and informed consent were approved by the Universidad de Antioquia’s Medical Research Institute and HUSVF Ethics Committees. All the experiments were performed in compliance with good clinical practice and good laboratory practice, in accordance with relevant guidelines and regulations of Universidad de Antioquia’s Medical Research Institute. All patients and HCs provided signed informed consent.

### Cytokines, autoantibodies, and CRP determination

To determine the serum cytokines IL-8, IL-6, IL-10, TNF-α, IL-1β, and IL12p70, the BD^TM^ Human Inflammatory CBA was used according to the manufacturer’s instructions.

IgM and IgG RF, as well as the IgG anti-CCP, were determined in the serum of patients by commercial ELISA (Quanta-Lite RF-IgM, Quanta-Lite RF-IgG and Quanta-Lite CCP3-IgG; Innova Diagnostics, San Diego, CA) according to the manufacturer’s instructions. A positive result for RF was >20 IU/mL and for anti-CCP was >10 IU/mL according to the commercial house. Results between 10 and <250 IU/mL and ≥250 IU/mL for these antibodies were arbitrarily considered for the definition of the anti-CCP^+^RF^+/−^ and anti-CCP^hi^RF^hi^ groups, respectively.

CRP quantification was performed by immunoturbimetry using the commercial kit Reagent Dimension Flex Quantitative Determination CRP Extended Range and the Dimension Clinical Chemistry Systems (both from McKesson, San Francisco, CA).

### Monocyte subset classification

The monocyte subsets were defined from total blood and SF by flow cytometry as we previously reported^19,53^: CD14++CD16− (classical), CD14++CD16+ (intermediate), and CD14 + CD16 ++ (non-classical) monocytes. Briefly, total blood was stained with anti-human CD45, CD14, CD16, and HLA-DR antibodies for 20 min at room temperature in darkness; fluorescence minus one (FMO) controls were performed for each antibody, and 100,000 cells were acquired immediately on the FACS Canto^TM^ II flow cytometer by using the FACS DIVA software (BD).

### Monocyte subsets immunophenotyping

Mononuclear cells from blood and SF were obtained by using a Ficoll density gradient as we previously described^19^. The expression of each molecule was evaluated by using specific antibodies against CCR2, CX3CR1, CD64, CD36, CD86, CD11b, CD32, FcμR, CD18, and HLA-DR on monocyte subsets (basic panel CD14, CD16, and HLA-DR). FMO controls were performed for each antibody by using the basic panel. Cells were blocked (DPBS plus 1% bovine serum albumin (BSA), 0.01% NaN_3_, and 10% inactivated FBS) and stained for 30 min at 4 °C, followed by two washes with washing buffer (DPBS plus 1% BSA and 0.01% NaN_3_). Immediately, 50,000 monocytes were acquired by flow cytometry.

### EVs characterization

Circulating and SF EVs were isolated and characterized as we previously described^19^ by using monoclonal antibodies against human CD41a-PE, CD45-PE-Cy7, CD105-BV421, CD33-PE, CD235a-APC, CD3-PE, CD19-V450, CD14FITC, CD16-BV450, HMGB1-APC, C1q-FITC, and HLA-DR-APC-Cy7. To measure EV-ICs, F(ab’)_2_ anti-IgG-Alexa Fluor-488 and anti-IgM-Alexa Fluor-647 fragments were used. To evaluate the presence of citrullinated peptides on EVs by flow cytometry, a specific primary antibody (rabbit polyclonal anti-human citrulline peptides antibody), and Alexa Fluor 647-labeled secondary antibody (anti-rabbit IgG H&L-Alexa Fluor 647-secondary antibody) were used. The EV size and count were evaluated with reference beads as we previously described^19^. Some EVs samples from patients with RA and HCs were evaluated through scanning transmission electron microscopy (STEM). For this purpose, the vesicles were fixed with 2.5% glutaraldehyde, deposited on copper-coated carbon STEM grids; dried in the grid at 40 °C, and contrasted with uranyl acetate and lead citrate. Finally, the samples were dried at room temperature and evaluated in the Tecnai G2 F20 microscope (FEI company, Hillsboro, OR). Images were recorded with an Ultra Scan 1000XP-P camera (Gatan, Inc, Pleasanton, CA).

### EV-ICs formation

EVs from plasma poor-platelets of eight patients with seropositive RA were obtained and frozen until use. For opsonization, approximately 1 × 10^6^ EVs were mixed and incubated with 15 µg/mL purified IgG (isolated from plasma of 16 RA seropositive patients by affinity chromatography) for 60 min at 37 °C. The unbound antibodies were washed away with 1 mL of DPBS at 17,000 *g* for 60 min. The EV- formation was assessed by flow cytometry (Figure S3).

### Monocyte isolation for culture with EVs and EV-ICs

Circulating monocytes were enriched by Rosette Sep, according to the manufacturer’s instructions. The purity of the CD14+ monocytes in all cases was greater than 90%. The binding and uptake of EVs and EV-ICs stained with 2 μM CFSE by monocyte subsets and phagocytes after 24 h of culture were performed at a ratio of 1:3 (cell: EVs) as reported^19^. To estimate the mean fluorescence intensity (MFI) of cells that internalized EVs, the samples were acquired on a flow cytometer in the presence or absence of 0.01% v/v trypan blue to quench the green fluorescence of surface-bound EVs.

Approximately 1.2 × 10^5^ monocytes were cultured as we previously described^54^. Briefly, the cells were adhered in RPMI-1640-GlutaMAX with 0.5% inactivated autologous serum-depleted of EVs (iSA-f)^19^. Then, the cells were incubated in RPMI-1640 with 10% iSA-f in the presence or absence of EVs and EV-ICs for 120 hours. The supernatants were collected and frozen at −20 °C until cytokine assessment by CBA.

### Data analysis

Comparisons among the RA groups’ patients and HCs were performed by Kruskal–Wallis test and Dunn’s *post-hoc* test (data as median ± interquartile range). Comparisons of more than one variable were performed by using ANOVA-II and Bonferroni *post-hoc* test (data as mean ± standard deviation). Statistical significance was set at p ≤ 0.05; *p ≤ 0.05, **p ≤ 0.01, ***p ≤ 0.001, and ****p ≤ 0.0001. Correlations were made by determining the Spearman’s rank coefficient with a 95% confidence interval. To illustrate the variations in size and content of EVs, t-SNE analysis was applied, with perplexity set to 100, and down sampling to 50,000; t-SNE plots were colored according to the expression of chosen channels by using ACCENSE 0.0.5-beta software^55^. The gating analyses, cells/EV frequencies, and MFI were estimated by using FlowJo 10.2 software. The statistical analysis was performed by using GraphPad Prism version 7.2 (GraphPad Soft-ware Inc., San Diego, CA). Multivariate discriminant analysis and PCA were performed by using Statgraphics X64 Centurion XVI (Statpoint Technologies, Warrenton, VA, USA).

## Electronic supplementary material

Supplementary Information

## Data Availability

The datasets generated during and/or analyzed during the current study are available from the corresponding author on reasonable request.
