# Supplementary material for: Extracellular vesicles are associated with the systemic inflammation of patients with seropositive rheumatoid arthritis
Source: Sci Rep. 2018 Dec 17;8:17917. doi: 10.1038/s41598-018-36335-x (PMC6297132; doi:10.1038/s41598-018-36335-x)
Supplement: Supplementary file 1 — Supplementary Information [file 41598_2018_36335_MOESM1_ESM.pdf]

Extracellular vesicles are associated with the systemic inflammation of patients with seropositive rheumatoid arthritis

Catalina Burbano[1,2], Mauricio Rojas[1,2], Carlos Muñoz-Vahos[3], Adriana Vanegas-García[3], Luis A. Correa[4], Gloria Vásquez[1], Diana Castaño[1,*].

[1] Grupo de Inmunología Celular e Inmunogenética, Instituto de Investigaciones Médicas, Facultad de Medicina, Universidad de Antioquia UdeA, Calle 70 No 52-21, Medellín, Colombia.

[2] Unidad de Citometría de Flujo, Sede de Investigación Universitaria, Universidad de Antioquia UdeA, Calle 70 No 52-21, Medellín, Colombia

[3]Sección de Reumatología. Hospital Universitario de San Vicente Fundación. Medellín, Colombia.

[4]Sección de Dermatología, Departamento de Medicina Interna, Facultad de Medicina, Universidad de Antioquia. Laboratorio Clínico VID, Obra de la Congregación Mariana, Medellín, Colombia

Supplementary Figures and Legends

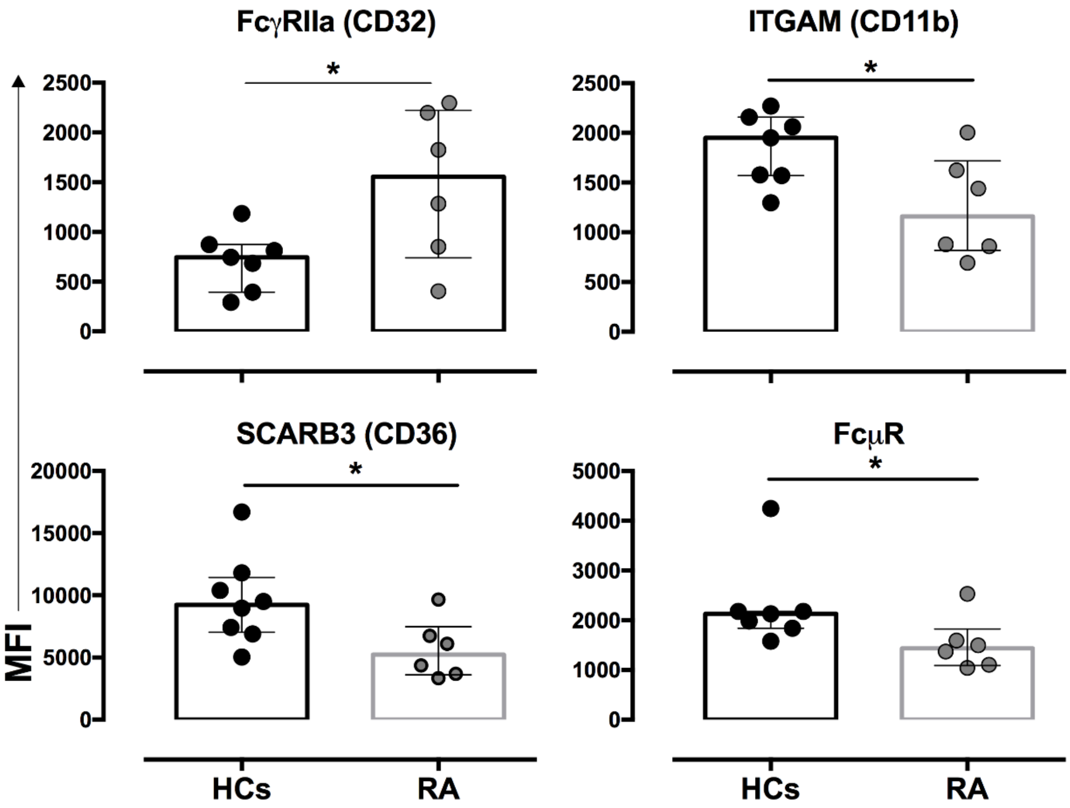

Figure S1. Mononuclear phagocytes from RA patients after 24 h of culture have higher expression of CD32 and decreases in CD11b, CD36, and FcμR relative to those of HCs. MFIs of CD32, CD11b, CD36, and FcμR on mononuclear phagocytes from HCs and seropositive RA patients cultured for 24 h. Comparisons between the groups were performed by using the Mann–Whitney test.

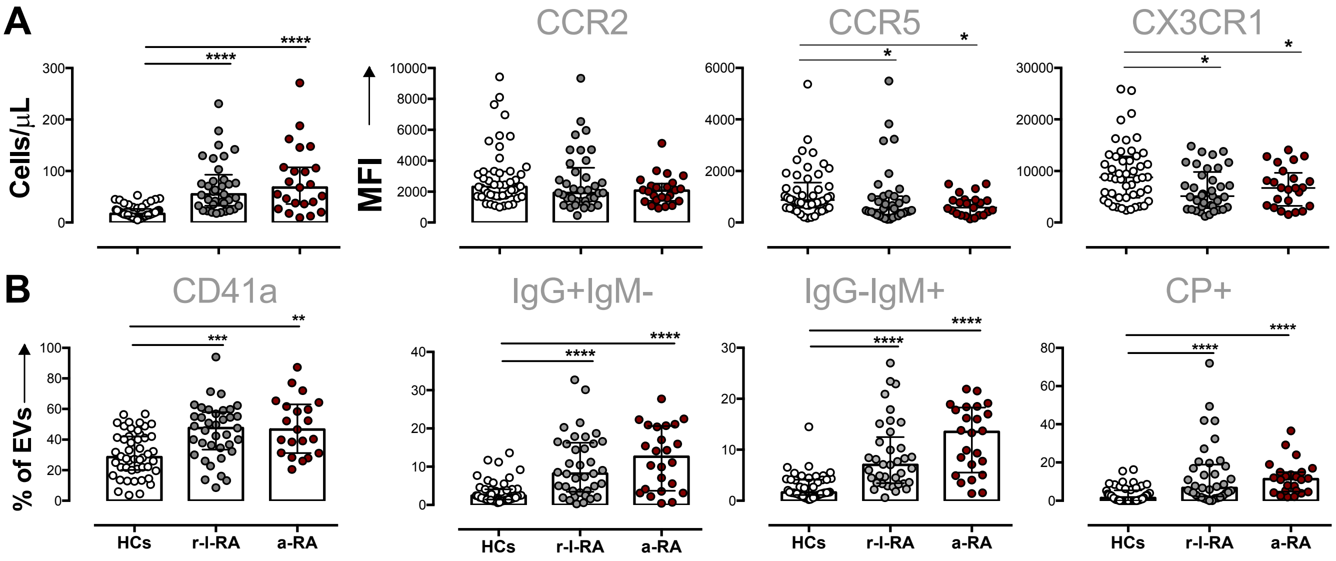

Figure S2. No differences were observed in intermediate monocytes and EVs between patients with RA in remission or with low activity versus patients with active disease. A. Absolute count and MFI of CCR2, CCR5, and CX3CR1 of intermediate monocytes from HCs (n = 40) and patients with RA: r-l-RA (remission and low activity; DAS28 ≤ 3.2, n = 36) and a-RA (moderate and high; DAS28 > 3.2, n = 24). **B**. Frequency of circulating EVs positive to CD41a, IgG+IgM-, IgG-IgM+ and CP+ from HCs and patients with RA: r-l-RA and a-RA. Comparisons among the groups were performed using the Kruskal–Wallis test followed by Dunn's post-hoc test.

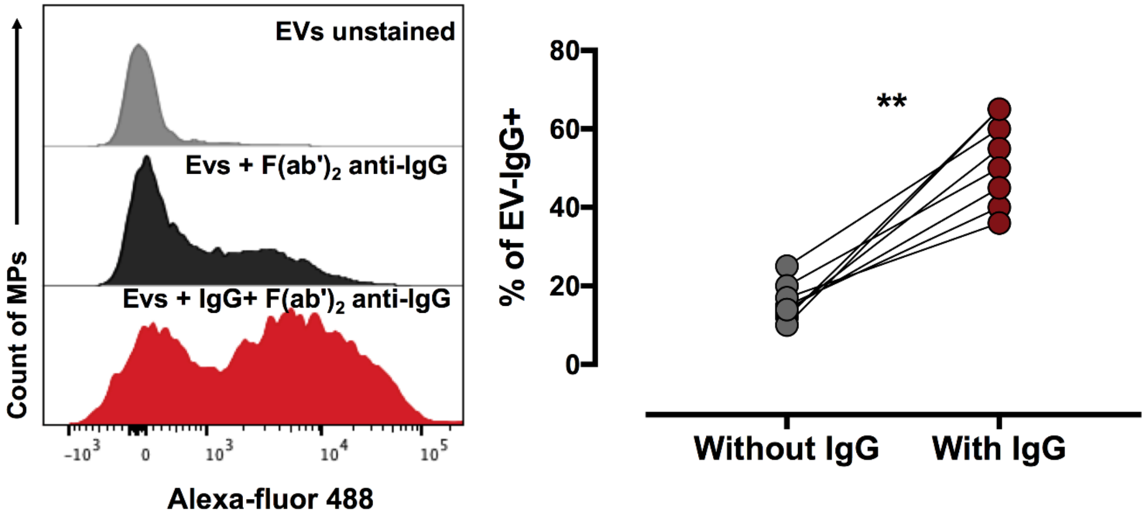

Figure S3. The frequency of IC formation by EVs (EV-ICs) increases after opsonization with purified IgG. Left. Representative histograms of EVs unstained (gray), EVs incubated without (black) or with (red) purified IgG from a patient with seropositive RA and staining with a F(ab')$_2$ anti-IgG fragment. **Right.** Frequency of EV-IgG+ incubated without or with purified IgG. Comparisons between the groups were performed by using the Wilcoxon signed-rank test (n = 8 seropositive RA patients).
